# Spinal motoneuron synaptic plasticity after axotomy in the absence of inducible nitric oxide synthase

**DOI:** 10.1186/1742-2094-7-31

**Published:** 2010-05-24

**Authors:** Amanda Emirandetti, Gustavo F Simões, Renata G Zanon, Alexandre LR Oliveira

**Affiliations:** 1 Department of Anatomy, Institute of Biology, University of Campinas (UNICAMP), CP 6109, CEP 13083-970, Campinas, SP, Brazil

## Abstract

**Background:**

Astrocytes play a major role in preserving and restoring structural and physiological integrity following injury to the nervous system. After peripheral axotomy, reactive gliosis propagates within adjacent spinal segments, influenced by the local synthesis of nitric oxide (NO). The present work investigated the importance of inducible nitric oxide synthase (iNOS) activity in acute and late glial responses after injury and in major histocompatibility complex class I (MHC I) expression and synaptic plasticity of inputs to lesioned alpha motoneurons.

**Methods:**

*In vivo *analyses were carried out using C57BL/6J-iNOS knockout (iNOS^-/-^) and C57BL/6J mice. Glial response after axotomy, glial MHC I expression, and the effects of axotomy on synaptic contacts were measured using immunohistochemistry and transmission electron microscopy. For this purpose, 2-month-old animals were sacrificed and fixed one or two weeks after unilateral sciatic nerve transection, and spinal cord sections were incubated with antibodies against classical MHC I, GFAP (glial fibrillary acidic protein - an astroglial marker), Iba-1 (an ionized calcium binding adaptor protein and a microglial marker) or synaptophysin (a presynaptic terminal marker). Western blotting analysis of MHC I and nNOS expression one week after lesion were also performed. The data were analyzed using a two-tailed Student's *t *test for parametric data or a two-tailed Mann-Whitney *U *test for nonparametric data.

**Results:**

A statistical difference was shown with respect to astrogliosis between strains at the different time points studied. Also, MHC I expression by iNOS^-/- ^microglial cells did not increase at one or two weeks after unilateral axotomy. There was a difference in synaptophysin expression reflecting synaptic elimination, in which iNOS^-/- ^mice displayed a decreased number of the inputs to alpha motoneurons, in comparison to that of C57BL/6J.

**Conclusion:**

The findings herein indicate that iNOS isoform activity influences MHC I expression by microglial cells one and two weeks after axotomy. This finding was associated with differences in astrogliosis, number of presynaptic terminals and synaptic covering of alpha motoneurons after lesioning in the mutant mice.

## Background

Nitric oxide (NO) is a gaseous free radical generated in most cells as a result of a diverse range of stimuli. This molecule may show protective effects in the nervous system, although pathologically elevated levels result in cytotoxicity. There are three major forms of enzyme that synthesize NO from L-arginine: the so-called NOS (nitric oxide synthases), with a 50-60% sequence homology between species [1]; neuronal (nNOS or NOS I) and endothelial (eNOS or NOS III) types, comprising the constitutive isoforms; and finally the inducible type (iNOS or NOS II). A fourth subtype of NOS (mtNOS) is an isoform of nNOS, and has been found in the inner mitochondrial membrane of several tissues including those of the liver, brain, heart and muscles [2,3].

The NO molecule has been implicated in several processes such as brain regulation [4,5], liver microcirculation [6], neuronal regeneration [7-9], neuronal damage [10], elimination of misdirected axons [11] and synaptic plasticity [12]. However, in recent years researchers have noted a dual role for NO, acting either as a pro-apoptotic mediator [13] or as an anti-apoptotic agent [14,15]. NO can have a protective effect on dorsal root ganglion (DRG) neurons *in vitro *via inhibition of Bax and caspases [16]. On the other hand, systemic NO inhibition by *Nω*-nitro-L-arginine (NOLA) or *N*-nitro-L-arginine methyl ester (L-NAME) results in delayed axonal degeneration after intraorbital optic nerve transection [17].

Peripheral nerve lesions, such as sciatic nerve transection, induce upregulation of all NOS isoforms, as demonstrated by NADPH-diaphorase histochemistry [18], NOS immunohistochemistry [19,20] and *in situ *hybridization [21]. Such increases in NOS expression result in enhanced expression of NO in the nerve microenvironment. Nevertheless, retrograde changes in response to peripheral axotomy also affect motoneuron cell bodies present in the spinal cord microenvironment. Secondarily to this, a prominent glial reaction develops in the spinal segments affected by a peripheral lesion, contributing to an intense rearrangement of synapses.

The mechanisms underlying neuron/neuron and neuron/glial communication after such lesioning remain poorly understood. Recently, the expression of the class I major histocompatibility complex (MHC I) was related to synaptic plasticity and to astrogliosis after peripheral nerve transection [22-26]. Interestingly, A/J mice, which present a greater axonal regeneration potential [27,28], also display more robust glial fibrillary acidic (GFAP) and ERM (ezrin-radixin-miosin) protein expression in spinal cord after axotomy, when compared to other strains of mice [24-26]. The Ca^2+^-calmodulin-independent isoform, iNOS, is expressed by astrocytes, macrophages and microglia following immunological or inflammatory stimulation [29-31]. This isoform has also been reported in other cells such as human hepatocytes [32] and rat vascular smooth muscle cells [33]. After axotomy, knockout mice lacking iNOS show slower Wallerian degeneration and fewer regenerating myelinated fibers [9]. Also, the absence of inducible nitric oxide at the site of the lesion can be related to subsequent neuropathic pain and decreased functional hind limb recovery. It has been suggested that astrocytes and microglia may be implicated in such an outcome [34,35].

Taking into account the profound acute changes in spinal cord circuits following a peripheral lesion, it is possible that NO may be involved in signaling pathways that induce MHC I upregulation by neurons and glia. Thus the present study investigated the process of synaptic plasticity after peripheral axotomy in the spinal cord of knockout mice lacking the inducible form of nitric oxide synthase (iNOS^-/-^). Different techniques (immunohistochemistry, western blotting and transmission electron microscopy) were used to investigate classical MHC I expression as well as glial responses following peripheral nerve injury. It is proposed that iNOS expression influences the outcome of the synaptic plasticity process in spinal cord, which is correlated with reduced MHC I expression.

## Methods

### Animals

For the present study, 30 6- to 8-week-old iNOS^-/- ^http://jaxmice.jax.org/strain/002596.html and 30 C57BL/6J age-matched wild type (WT) mice (~25.0 g body weight) were used. The animals were obtained from the Multidisciplinary Center for Biological Investigation (CEMIB/Unicamp) and were housed with a 12 h light/dark cycle and free access to food and water. The study was approved by the Institutional Committee for Ethics in Animal Experimentation (CEEA/IB/Unicamp, proc. 1172-1), and all the experiments were carried out according to the guidelines of the Brazilian College for Animal Experimentation (COBEA). The mice from both strains were subjected to unilateral sciatic nerve transection as described below. The unlesioned sides were used as controls.

### Surgical procedures and tissue preparation

The mice were anesthetized with a mixture of Kensol (Xylazin, König, 10 mg/Kg) and Vetaset (Cetamin, Fort Dodge, 50 mg/Kg; 1:1, 0.12 ml/25 g, i.p.), and subjected to left sciatic nerve transection at level of the obturator tendon. A 2-mm-long segment of the distal stump was removed to avoid regeneration. Muscle and skin planes were sutured, and the animals allowed to survive for 1 or 2 weeks. All mice were sacrificed using an overdose of anesthetic, subjected to transcardial perfusion with 0.1 M PBS (phosphate buffered saline, 20 ml, pH 7.4) and fixed either in 10% formaldehyde in PB (phosphate buffer) for immunohistochemistry or with Karnovsky fixative (2.5% glutaraldehyde and 1.0% paraformaldehyde in 0.1 M phosphate buffer pH 7.4) for electron microscopy. Spinal cord segments L4-L6 were dissected out and either frozen (for immunohistochemistry), embedded in resin (for electron microscopy) or processed for western blotting analysis (non-fixed tissue).

### Immunohistochemistry

**Figure 1:**
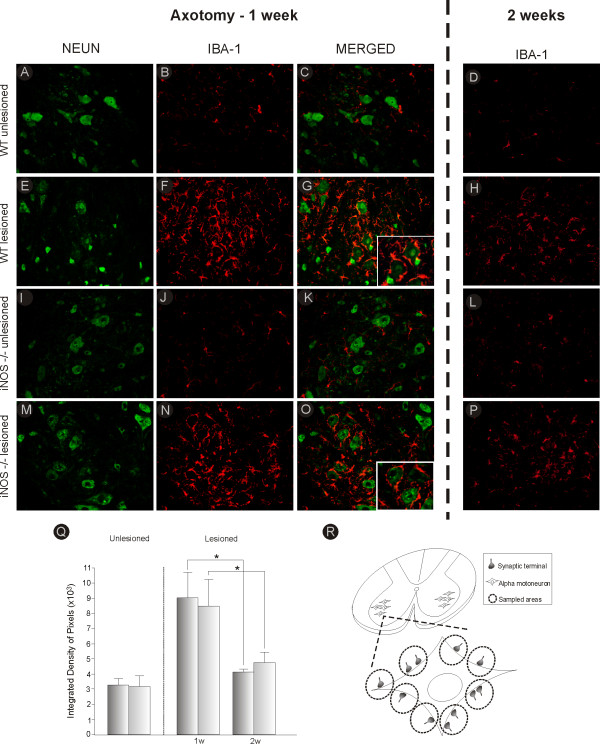
**Iba-1 and NeuN double-labeling in C57BL6/J and iNOS^-/- ^mice, one and two weeks after axotomy**. Observe the strong upregulation of Iba-1 labeling in C57BL6/J (WT, **F**) and iNOS^-/- ^**(N) **one week after lesion, as compared to the unlesioned side **(B and J)**. Two weeks after axotomy, the microglial reaction decreased **(H and P)**. The microglia presented in close apposition to axotomized motoneurons **(G and O)**. **(A and I) **NeuN labeling on the contralateral side of C57BL6/J and iNOS^-/- ^mice, respectively. **(E and M) **NeuN labeling on the ipsilateral side of C57BL6/J and iNOS^-/- ^mice, respectively. **(C and K) **Iba-1 and NeuN double-labeling in the unlesioned side of C57BL6/J and iNOS^-/- ^mice. **(D and L) **Iba-1 labeling on the unlesioned side of C57BL6/J and iNOS^-/- ^mice, two weeks after axotomy. **(Q) **Graph representing quantification of the integrated density of pixels (×10^3^) for Iba-1 immunohistochemistry in the neuropil of the unlesioned and lesioned sides, one (1 w) and two (2 w) weeks after axotomy. * = p < 0.05 (Mann-Whitney *U *test). Scale bar = 50 μm. **(R) **Schematic representation of the sciatic nerve pool in the ventral horn of spinal cord. One motoneuron is shown in detail with apposed pre-synaptic terminals. The dashed circles represent areas where measurements of the integrated density of pixels were performed.

Lumbar spinal cords from animals sacrificed one or two weeks after axotomy were frozen at -40°C for cryostat sectioning (12 μm thickness). In the primary incubation, the following antisera were used: goat anti-GFAP (1:500, Santa Cruz Biotechnology, USA), rabbit anti-Iba-1 (antibody against the ionized calcium binding adaptor protein, 1:1000, Wako, USA [36]), rat anti-classical MHC I (ER-Hr52, which recognizes H2-K^b ^and H2-D^b^, the two classical MHC I in C57BL6/J mice, 1:200, BMA Biomedicals, Switzerland), mouse anti-nNOS (1:200, BD Biosciences, USA), mouse anti-iNOS (1:200, Santa Cruz Biotechnology, USA), mouse anti-NeuN (antibody against neuronal nuclear antigen, 1:600, Chemicon, USA) or rabbit anti-synaptophysin (an antibody against a protein present in the membrane of synaptic vesicles 1:200, Dako, Denmark). The antibodies were diluted in a solution containing BSA and Triton x-100 in 0.01 M PBS and the slides were post-fixed in cold acetone for 30 seconds prior to the classical MHC I incubation; the sections were incubated overnight at 4°C in a moist chamber. After rinsing in 0.01 M PBS, sections were incubated with Cy3, Cy2 or FICT-conjugated secondary antisera (1:250, Jackson ImmunoResearch) for 45 min in a moist chamber at room temperature. The slides were then rinsed in PBS, mounted in a glycerol/PBS (3:1) mixture and observed using an inverted fluorescence microscope (Eclipse TS100, Nikon) equipped with a high resolution camera (DXM 1200F, Nikon). For quantitative measurements, the lesioned segments were first identified by the presence of decreased synaptophysin immunolabeling in the motoneuron microenvironment, combined with an increased glial reaction. Three alternate sections (ipsi- and contralateral sides of the spinal cord) from each animal (n = 10 for each group) were then used to capture images from the ventral horn at a final magnification of x200, always maintaining all settings unchanged. Because of the reduced area occupied by alpha motoneurons in the dorsolateral region of the ventral horn and because several sections were used to perform immunohistochemistry, 10 mice per group were used. However, only 5 animals per group were allocated for the quantitative analysis. Quantification was performed around the axotomized motoneurons using the enhance contrast and density slicing feature of IMAGEJ software (version 1.33u, National Institute of Health, USA). The integrated density of pixels (sum of the gray values of each pixel in a determined area) around each motoneuron identified in the lateral motor nucleus was measured in eight circular areas from each side, as shown in Figure 1R. This quantification method measures the intensity of fluorescence in a given image, and has been used in previous studies [23-26]. A lesioned/unlesioned ratio for the integrated density of pixels was calculated for each section (axotomy groups) and then a mean value was calculated for each spinal cord. The data are represented as mean ± standard deviation (SD).

### Western blotting

For quantification of MHC class I and nNOS expressions, 3-mm portions of lumbar spinal cord (L4-L6 segments) were cut from the right (contralateral) and left (ipsilateral to the axotomy) sides. All specimens were sonicated in Rippa buffer protein extraction medium (150 mM NaCl, 50 mM Tris pH 8.0, 1 mM PMSF, 1 mM EDTA, 0.5% Na-deoxycholate acid, 0.1% SDS and 1% Triton X-100) for 1 min. Total protein concentration was measured using Bio-Rad Bradford's protein assay.

Western blotting was performed after submitting 100 μg (MHC class I) or 40 μg (nNOS) of protein extract from each tissue sample to electrophoresis in 10% (MHC I) or 12.5% (nNOS) polyacrylamide gels under reducing conditions, and electric transfer to nitrocellulose membranes (Hybond-ECL; Amersham Biosciences, Chalfont St. Giles, United Kingdom). The membranes were blocked for 1 h with 1% or 5% nonfat milk powder in Tris buffered saline plus 0.2% Tween 20 (TBS-T) at room temperature, with agitation. Rat anti-MHC class I (ER-Hr52 monoclonal, 1:500, Peninsula, USA), mouse anti-nNOS (monoclonal, 1:500, Santa Cruz Biotechnology, USA) or rabbit anti-beta actin (polyclonal, 1:5000, Abcam, USA) antibodies were diluted in 0.1 or 1% nonfat milk powder in TBS-T and incubated overnight at 4°C. After the primary antisera, three TBS-T washes were carried out and either HRP-conjugated rabbit anti-rat, rabbit anti-mouse or goat anti-rabbit IgG antibody (1:2500, in TBS-T, Zymed Laboratories, USA) - according to the host of the primary antibody - was added for 1 h at room temperature, with agitation. After a further set of washes, detection of the labeling was achieved by chemiluminescence (Perkin-Elmer, Waltham, USA). Band intensity was determined by densitometry using ImageJ Software (version 1.33u, National Institute of Health, USA).

### Electron microscopy

Lumbar spinal cords (n = 5 for each group) were dissected out and stored overnight in fixative at 4°C. The specimens were then trimmed, osmicated, dehydrated, and embedded in Durcupan (Fluka). Ultrathin sections from the L4-L6 segments were collected on formvar-coated copper grids, counterstained with 4% uranyl acetate and lead citrate, and examined under a transmission electron microscope operating at 60 KV (Leo 906, ZEISS). Neurons with large cell bodies (~35 μm in diameter), found in the sciatic motoneuron pool and cut in the nuclear plane, were identified as α-motoneurons by the presence of C-type nerve terminals and chromatolysis (axotomized side). The cell surfaces were then sequentially digitalized at a magnification of 12.930X with a video camera connected to a computer, and the images mounted together using vectorial software. Synaptic terminals apposing the motoneuron somata were identified and their numbers per 100 μm of cell membrane, as well as the membrane covering of all the terminals (calculated in percent of membrane length), determined using the measurement tool of Image Tool software (Version 3.0, The University of Texas Health Center, USA). The terminals were typed as F-type (with flattened synaptic vesicles), S-type (with spherical synaptic vesicles) or C-type (with a sub synaptic cistern), according to the procedure described by Conradi [37]. The distance between consecutive nerve terminals covering the motoneurons was also determined. A total of 30 sciatic α-motoneurons (two neurons per animal in three groups of five animals: iNOS^-/- ^axotomized, WT axotomized and WT unlesioned) were quantified. The data were represented as the mean ± standard deviation (SD).

### Statistical analysis

The data were analyzed using a two-tailed Student's *t *test for parametric data or a two-tailed Mann-Whitney *U *test for nonparametric data at p < 0.05 (*), p < 0.01 (**), and p < 0.001 (***). The statistical analyses and graph plotting were done by using tools from a GraphPad Prism4 program.

## Results

### Glial reaction and classical MHC I spinal expression in the absence of inducible nitric oxide one and two weeks after lesioning

The effects of sciatic nerve transection on microglial reaction were assessed with quantitative measurements of immunoreactivity around the spinal cord motor nucleus one and two weeks after lesion, using anti-Iba-1 antiserum (antibody against the ionized calcium binding adaptor protein, a microglial marker [36]; Figure 1). Microglial cells were not reactive in the areas surrounding NeuN-labeled motoneurons on the unlesioned side (Figures. 1C and 1K) and quantitative analysis showed no statistical differences between C57BL6/J and iNOS^-/- ^mice (C57BL6/J, 3.13 × 10^3 ^± 0.62; iNOS^-/-^, 3.20 ± 0.47; Figure 1Q), indicating that both strains displayed equivalent basal expression of the referred protein (also seen in Figures 1B and 1J). However, there was an increase in Iba-1 expression on the lesioned side of both strains one and two weeks after axotomy (Figures 1F, N, H and 1P). It is important to emphasize the close relationship between the axotomized α-motoneurons and the reactive microglia, as depicted in figures 1G and 1O, respectively. The integrated density of pixels in the lesioned side was also determined and showed statistical differences between the two studied time points in both strains (C57BL6/J one week, 8.43 × 10^3 ^± 0.47; two weeks, 4.70 × 10^3 ^± 0.73, p < 0.05 and iNOS^-/- ^one week, 8.99 × 10^3 ^± 1.48; two weeks, 4.08 × 10^3 ^± 0.21, p < 0.05, Figure 1Q).

**Figure 2:**
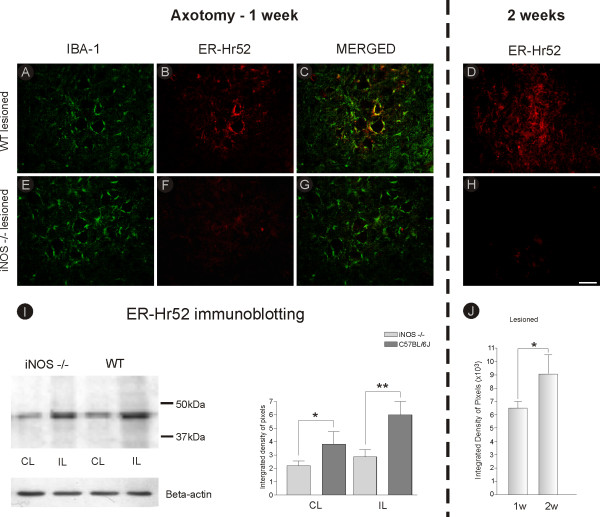
**Classical MHC I and Iba-1 double-staining in C57BL6/J and iNOS^-/- ^mice, one and two weeks after axotomy**. Observe the significantly stronger upregulation of MHC I (ER-Hr52) labeling in C57BL6/J (WT) mice one week after lesion **(B)**, which is co-localized with microglial Iba-1 positive cells **(C)**. The lesioned side **(F) **of the spinal cord of iNOS^-/- ^mice shows almost no labeling, **(A and E) **Iba-1 labeling on the lesioned side of C57BL6/J and iNOS^-/- ^mice, respectively. Two weeks after lesioning, ER-HR52 immunolabeling is even stronger than in the acute phase **(D)**. **(H) **No MHC I labeling was seen in the ventral horn of iNOS^-/- ^mice, two weeks after sciatic transection. **(I)**. Western blotting analysis of MHC I expression in spinal cord ventral horn ipsi- and contralateral to axotomy. Note the upregulation of MHC I in wild type mice after lesion, which is not observed in iNOS^-/- ^animals. β-Actin was used as sample loading control. IL = ipsilateral; CL = contralateral. * = p < 0.05; ** = p < 0.01 (Mann-Whitney *U *test). **(J) **Graph representing quantification of the integrated density of pixels for MHC I immunolabeling in the neuropil adjacent to large motoneurons, one (1 w) and two (2 w) weeks after axotomy. * = p < 0.05, Student *t *test. Scale bar = 50 μm.

In order to analyze expression of classical MHC I in ventral horn, Iba-1 and MHC I double labeling was performed. Such immunolabeling also assured that the spinal segments analyzed contained lesioned α-motoneurons (Figure 2). Lesioning did not result in any significant increase of MHC I expression on the unlesioned side (not shown). However, at both one and two weeks after axotomy the C57BL6/J mice showed significant upregulation of MHC I protein in the surroundings of the motoneurons on the lesioned side (Figures 2B and 2D). This difference in MHC I expression by wild type microglial cells at different time points after lesioning is shown in Figure 2J (C57BL6/J one week, 6.51 × 10^3 ^± 0.49; two weeks, 9.06 × 10^3 ^± 1.43, p < 0.05). Interestingly, the elevated expression of MHC I in wild type mice co-localized with Iba-1 labeling (Figure 2C). Also, MHC I expression in iNOS knockout mice was not increased at either 7 or 21 days after axotomy (Figures 2F and 2H), indicating that the absence of MHC I expression in iNOS^-/- ^mice does not represent a delayed response but rather a collateral effect of the gene knockout. Western blot analysis confirmed the data obtained one week after lesioning and is depicted in Figure 2I (C57BL6/J 3.81 ± 2.05; iNOS^-/- ^2.19 ± 0.81, p < 0.05, contralateral; C57BL6/J 5.98 ± 2.23; iNOS^-/- ^2.85 ± 1.14; p < 0.01, ipsilateral).

**Figure 3:**
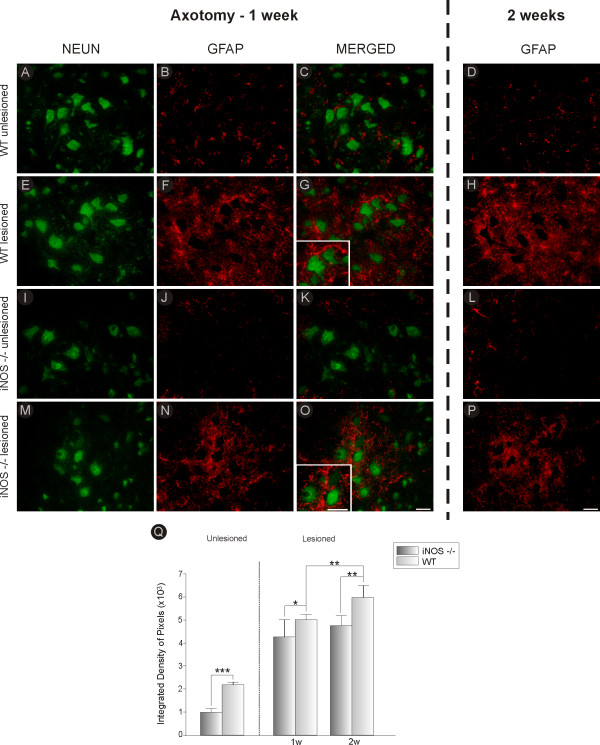
**GFAP and NeuN double-labeling in C57BL6/J and iNOS^-/- ^mice, one and two weeks after axotomy**. Observe the strong upregulation of GFAP labeling in C57BL6/J (WT, **F**) and iNOS^-/- ^**(N) **one week after lesion, as compared to the unlesioned side **(B and J)**. The iNOS^-/- ^unlesioned side **(J) **shows milder basal astroglial labeling as compared to control mice. After lesioning the astroglial reaction presented a diffuse distribution surrounding the axotomized pool of motoneurons in WT mice **(G)**, and was more concentrated around the motoneurons in iNOS-deficient mice **(O)**. **(A and I) **NeuN labeling on the contralateral side of C57BL6/J and iNOS^-/- ^mice, respectively. **(E and M) **NeuN labeling on the ipsilateral side of C57BL6/J and iNOS^-/- ^mice, respectively. **(C and K) **GFAP and NeuN double-labeling on the unlesioned side of C57BL6/J and iNOS^-/- ^mice. **(H and P) **GFAP labeling in C57BL6/J and iNOS^-/- ^mice, two weeks after axotomy, lesioned side. **(D and L) **GFAP labeling in C57BL6/J and iNOS^-/- ^mice, two weeks after axotomy, unlesioned side. **(Q) **Graph representing quantification of the integrated density of pixels for GFAP in the neuropil of the unlesioned and lesioned sides, one (1 w) and two weeks (2 w) after axotomy. * = p < 0.05, ** = p < 0.01 and *** = p < 0.001 (Student *t *test). Scale bar = 50 μm.

**Figure 4:**
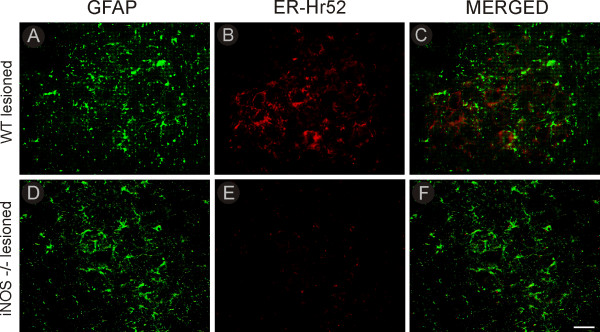
**Classical MHC I and GFAP double-staining in C57BL6/J and iNOS^-/- ^mice, one week after axotomy**. Observe that GFAP labeling in C57BL6/J (WT) mice is not co-localized with MHC I- (ER-Hr52) positive cells **(C)**. Again, axotomized iNOS^-/- ^mice did not express classical MHC I **(E)**. **(A and D) **GFAP labeling on the lesioned side of C57BL6/J and iNOS^-/- ^mice, respectively. Scale bar = 50 μm.

The level of astroglial reaction one and two weeks after axotomy was also studied in iNOS^-/- ^and WT mice by measuring immunostaining in the neuropil adjacent to lesioned NeuN-labeled motoneurons (Figure 3). To determine whether astrocytes could express classical MHC I, double labeling was performed using antibodies against GFAP and MHC I (Figure 4). As shown in Figure 4C, no significant co-localization was obtained.

**Figure 5:**
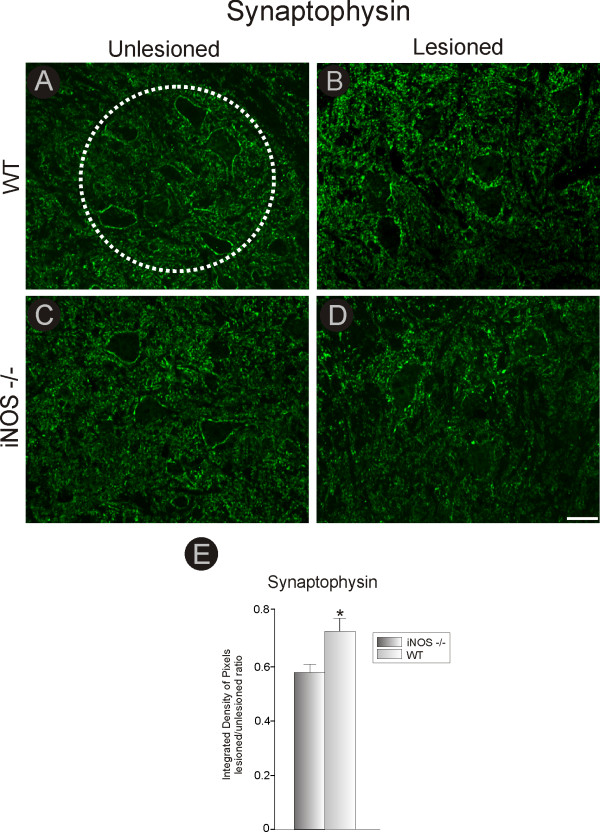
**Synaptophysin immunostaining in C57BL6/J and iNOS^-/- ^mice, one week after unilateral axotomy**. Note that one week after lesioning, there was a stronger decrease in labeling especially in the areas surrounding the motoneurons. This decrease was more intense in iNOS^-/- ^mice **(D) **than in C57BL6/J mice **(B)**. **(A and C) **Unlesioned side of C57BL6/J and iNOS^-/- ^mice, respectively. The dashed circle indicates the motor nucleus containing the alpha motoneurons. **(E) **Graph representing quantification of the integrated density of pixels in the neuropil adjacent to large motoneurons. * = p < 0.05 (Student *t *test). Scale bar = 50 μm.

There was a statistically significant difference in basal expression of GFAP between the two strains (as shown in Figures 3B and 3J). C57BL6/J mice displayed stronger basal labeling for GFAP compared to iNOS^-/- ^mice (C57BL6/J, 2.169 × 10^3 ^± 0.11; iNOS^-/-^, 0.98 × 10^3 ^± 0.14, p < 0.001; Figure 5Q). However, there was upregulation of GFAP expression one week (C57BL6/J, 5.01 × 10^3 ^± 0.22; iNOS^-/-^, 4.24 × 10^3 ^± 0.80, p < 0.05) and two weeks (C57BL6/J, 6.01 × 10^3 ^± 0.49; iNOS^-/-^, 4.76 × 10^3 ^± 0.44, p < 0.01) after sciatic nerve transection (Figure 3Q). GFAP labeling was more robust in iNOS^-/- ^mice 14 days after nerve lesion, as compared to one week post axotomy (p < 0.01, Figure 3Q). It is important to emphasize that the astroglial reaction presented a diffuse pattern in normal mice (Figure 3F and 3H), whereas it was restricted to the surroundings of the sciatic motoneuron pool in iNOS deficient mice (Figure 3N and 3P).

### Loss of inputs to axotomized iNOS^-/- ^spinal α-motoneurons is shown by a decrease in synaptophysin labeling

To analyze changes in synaptic activity resulting from axotomy, quantitative measurements of the expression of the synaptic protein synaptophysin were made for the sciatic motor nuclei of iNOS^-/- ^and C57BL6/J mice. Only large motoneurons present in the dorsolateral nucleus, which supplies the distal hind limb muscles, were considered for analysis. Figures 5A and 5B show similar synaptophysin immunoreactivity in ventral horn of WT and iNOS-deficient mice (Figure 5C). In both cases, a clear decrease in synaptophysin labeling occurred in the motor nuclei on the lesioned side, although this reduction was significantly greater in iNOS^-/- ^as compared to C57BL6/J mice (C57BL6/J, 0.72 ± 0.05; iNOS^-/-^, 0.53 ± 0.03, p < 0.05, lesioned/unlesioned side ratio, Figure 5E). The scheme shown in Figure 5F indicates the circular areas (100 μm^2 ^each) used for quantification of immunofluorescence. The differential degree of synaptic elimination observed by immunohistochemistry was further assessed using transmission electron microscopy, and these results are described below.

### Synaptic elimination, glial reaction and MHC expression are not influenced by neuronal nitric oxide synthase isoform (nNOS)

**Figure 6:**
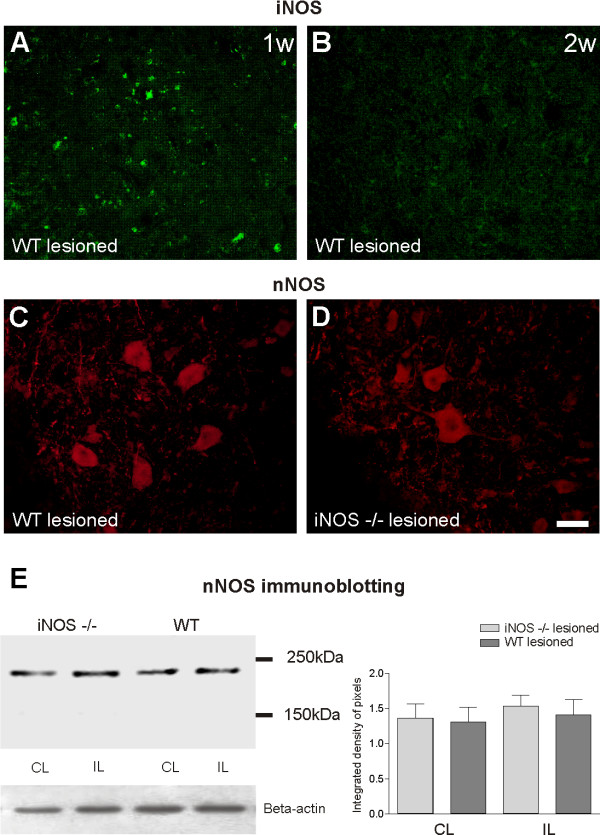
**iNOS and nNOS labeling in C57BL6/J and iNOS^-/- ^mice, one and two weeks after unilateral axotomy**. **(A) **positive iNOS labeling within the motor nucleus in C57BL6/J (WT) one week after axotomy (1 w). The lesioned side **(B) **of the spinal cord of WT mice shows no labeling two weeks after axotomy (2 w). **(C and D) **nNOS immunolabeling in C57BL6/J and iNOS^-/- ^ventral horn one week after lesioning, respectively. **(E) **Western blot analysis of nNOS expression in iNOS knockout mice and WT mice. Observe the absence of statistically significant nNOS upregulation after peripheral axotomy as well as the similar expression of such protein in both strains. β-Actin was used as sample loading control. IL = ipsilateral; CL = contralateral. Scale bar = 50 μm.

An important question to answer was whether the glial and neuronal events in response to sciatic nerve transection could be related to differential neuronal expression of nNOS in C57BL6/J mice after axotomy. For this purpose, immunohistochemistry using anti-nNOs antibody was performed in iNOS^-/- ^and C57BL6/J spinal cords (Figure 6). nNOS immunolabeling was observed within neurons throughout the spinal cord, including large motoneurons in both wild type and iNOS knockout mice (Figures 6C and 6D, respectively). Western blot analysis confirmed the immunolabeling results obtained *in situ*, and indicated that axotomy did not result in statistically significant nNOS activity upregulation (Figure 6E). Figure 6A shows iNOS expression by C57BL6/J glial cells one week (1 w) after transection. Such up-regulation was not detectable on the lesioned side, two weeks (2 w) after axotomy.

### Faster synaptic elimination in iNOS-deficient mice after lesioning (Figures 7, 8 and 9)

**Figure 7:**
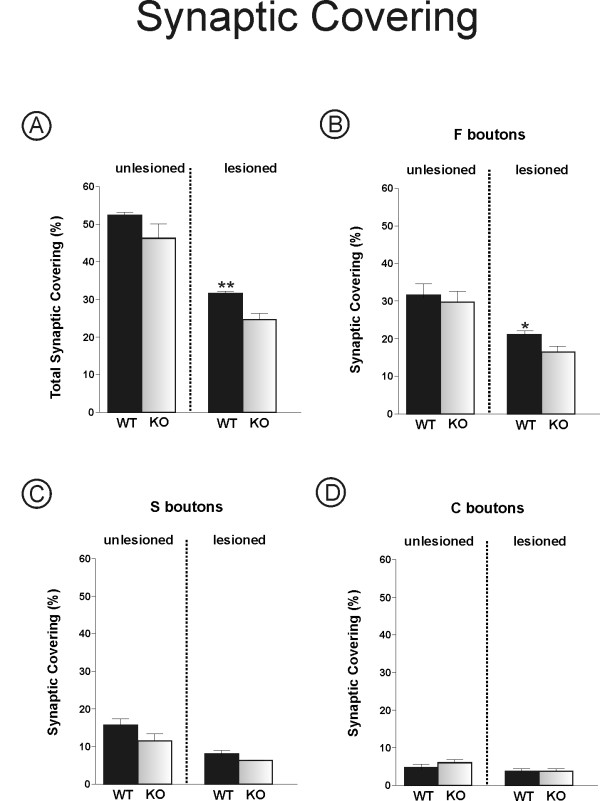
**Synaptic covering obtained by a detailed analysis of inputs in apposition to the surface of sciatic motoneurons**. **(A) **Percentage of absolute retraction of nerve terminals from apposition to the postsynaptic membrane in the different mice strains. One week after axotomy, a significant loss of covering can be seen in iNOS^-/- ^mice as compared to C57BL6/J mice. **(B - D) **Percentage of synaptic covering by F-, S- and C-terminals on the unlesioned and lesioned sides. Note that after injury the iNOS^-/- ^mice presented a greater loss of F- type terminals than the WT mice. * = p < 0.05 and ** = p < 0.01 (Mann-Whitney *U *test).

**Figure 8:**
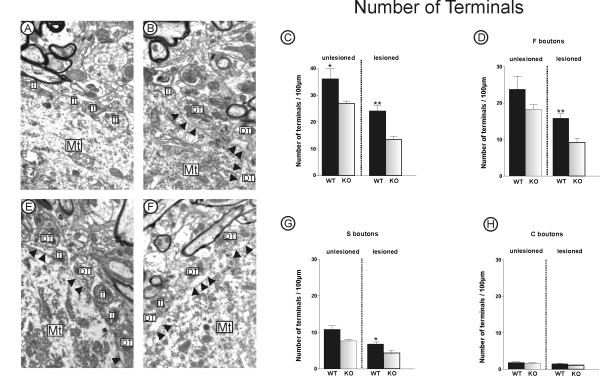
**Quantitative ultrastructural analyses of the number of synaptic terminals apposed to the surface of motoneurons after axotomy**. **(A and E) **Representative micrographs of the surface of unlesioned motoneurons from C57BL6/J and iNOS^-/- ^mice strains, respectively. One week after peripheral lesion, C57BL6/J mice show a few synaptic terminals detached from the surface of lesioned motoneurons **(B)**. Such detachment occurred to a greater degree in the ventral horn of the iNOS knockout strain **(F)**. Observe the comparatively lower degree of synaptic covering in iNOS^-/- ^mice as compared to C57BL6/J mice. DT = detached terminal, T = apposed terminal, Mt = motoneuron. The black arrows indicate the location of motoneuron membrane surfaces from which synaptic terminals were detached. **(C) **Graph of the total number of synaptic terminals per 100 μm of motoneuron membrane in lesioned and unlesioned neurons. Observe that unlesioned iNOS^-/- ^mice show greater synaptic elimination as compared to WT. This difference is even higher after axotomy. **(F, G and H) **Graphs of F-, S- and C-terminal numbers per 100 μm of motoneuron membranes, respectively. A greater loss of F-type terminals can be seen one week after axotomy in iNOS^-/- ^mice. * = p < 0.05 and ** = p < 0.01 (Mann-Whitney *U *test). Scale bar = 1 μm.

**Figure 9:**
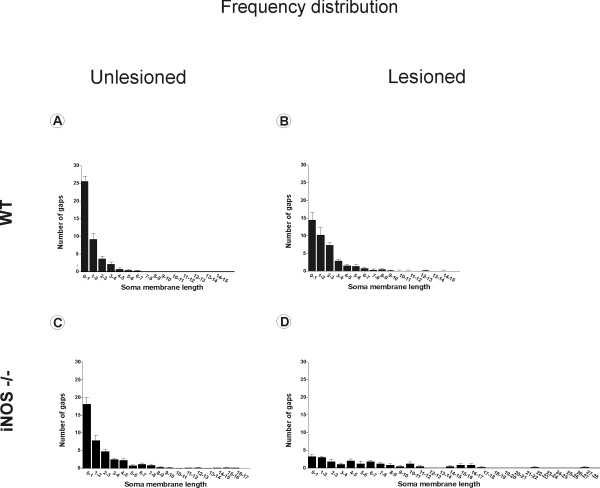
**Graphs showing frequency distributions of gaps between terminals along the motoneuron membrane**. The general gap distribution was retained in both strains one week after lesion indicating that, apart from differences regarding number and covering of terminals, synaptic elimination occurred in such a way that clusters of boutons were preserved. **(A and B) **Graph of the number of gaps between apposed terminals per length of membrane (μm) of C57BL6/J (WT) mice on the unlesioned and lesioned sides, respectively. **(C and D) **Graph of the number of gaps between apposed terminals per length of membrane (μm) of iNOS^-/- ^mice on the unlesioned and lesioned sides, respectively.

To better understand the different processes involved during synaptic elimination in iNOS-deficient and WT mice, a thorough ultrastructural analysis of inputs to α-motoneurons in the sciatic nerve pool was carried out. The general ultrastructure appeared identical for both mice strains studied. The total synaptic covering, which represents the motoneuron body surface in contact with presynaptic terminals, revealed the same general density of synapses in unlesioned iNOS^-/- ^and C57BL6/J mice, as shown in Figure 7A (C57BL6/J, 52.43% ± 0.74%; iNOS^-/-^, 46.28% ± 4.38%). However, one week after axotomy, the synaptic elimination process differed significantly when the strains were compared (C57BL6/J, 31.63% ± 0.57%; iNOS^-/-^, 24.61% ± 1.69%, p < 0.01). Moreover, total counts of terminals apposed to the motoneuron surface revealed a smaller number of boutons per 100 μm on the right side in the absence of iNOS (C57BL6/J, 36.08 ± 3.87; iNOS^-/-^, 26.89 ± 0.93, p < 0.05, Figure 8C). These values were even lower after axotomy (C57BL6/J, 22.58 ± 1.39; iNOS^-/-^, 13.43 ± 1.27, p < 0.01, Figure 8C). The results described above were further investigated under TEM in order to determine whether there was any qualitative difference in type of preserved inputs in the two referred strains. As described earlier [37], there are two main categories of synaptic terminals on motoneurons, which can be typed according to the shape of their synaptic vesicles. Type S terminals, with spherical vesicles, mostly contain the excitatory neurotransmitter glutamate, although a small proportion of large S terminals are cholinergic (C) [38,39]. When observed under TEM, the F terminals contain flattened or pleomorphic vesicles filled with glycine and/or GABA [40,41], and work as inhibitory inputs. Qualitative analysis of F, S and C boutons, presented in Figures 7 and 8, reveals that a greater number of F terminals were preserved in apposition to sciatic motoneurons in C57BL6/J mice as compared to iNOS^-/- ^mice (C57BL6/J, 15.12 ± 1.18; iNOS^-/-^, 8.78 ± 1.00, p < 0.01, Figure 8D) one week after cutting the sciatic nerve. S terminals were preserved to a smaller degree in normal mice (C57BL6/J, 6.27 ± 1.52; iNOS^-/-^, 3.99 ± 0.68, p < 0.05, Figure 8G). However, in terms of F synaptic covering, C57BL6/J mice displayed the greatest values after transection, as shown in Figure 7B (C57BL6/J, 20.79% ± 2.71%; iNOS^-/-^, 16.13% ± 1.64%, p < 0.05).

Figure 9A represents the normal pattern of terminal distribution along the motoneuron surface in WT mice. After axotomy, the gaps between clusters of boutons tended to increase due to selective retraction of inputs, as shown in Figure 9B. However, in the absence of iNOS, the number of gaps was decreased (Figure 9C) as a consequence of a diminished number of basal terminals characteristic of this strain, as previously shown in Figure 9A. The remaining terminals were usually close together, so that the general pattern of gap distribution was maintained. One week after axotomy, iNOS^-/- ^mice displayed a disturbed terminal distribution pattern, represented by a smaller number of gaps. In addition it was possible to find much longer gaps in the iNOS^-/- ^mice when compared to the C57BL6/J mice (between 16 and 30 μm long), indicating that the transgenic mice developed a stronger reaction to the peripheral lesion.

On the whole, these results with respect to synaptology may be interpreted to indicate that the acute response to injury is milder in C57BL6/J mice than in mice lacking inducible nitric oxide synthase, both in terms of synaptic retraction and terminal apposition surface.

## Discussion

It has been reported that neurons and glial cells in the CNS can express MHC class I complex in response to different stimuli, including neurodegenerative diseases such as Huntington's disease, Parkinson's disease, autism and schizophrenia [42,43], and also following peripheral nerve axotomy [44,24,26]. By demonstrating that expression of this immune molecule occurs within the CNS, such findings challenged the prevailing view that the brain is "immunoprivileged" [45,46]. This process is not only restricted to MHC I molecules, but is also valid for C1q, the initiating protein in the classical complement cascade, which mediates CNS synapse elimination during development [47].

Lindå *et al. *[44] used *in situ *hybridization techniques to show that motoneurons express β2-microglobulin mRNA (a co-subunit of the MHC class I complex) in response to sciatic nerve transection. In addition, a major contribution to identify putative signaling molecules in synaptic plasticity events was provided by Huh *et al. *[48], who demonstrated that functional expression of immune-recognition MHC class I molecules is linked to the process of synaptic elimination in the developing CNS.

More recently, Oliveira *et al. *[23] proposed a role for classical MHC I molecules in the mechanisms of synaptic plasticity after peripheral nerve axotomy. Using β2-microglobulin- and TAP I- (required for loading peptides onto the MHC I in the endoplasmic reticulum) knockout mice, they found that such immune molecules were involved in maintenance of inhibitory inputs after lesioning, leading to a significant detachment of glycine/GABA-containing synapses from the cell bodies of transected motoneurons. In addition, Goddard *et al. *[49] showed that MHC I proteins are co-localized postsynaptically with PSD-95 in dendrites of hippocampal neuronal cells. Earlier studies also showed that neurons express the MHC I complex, not only after axotomy [50,51], but also during the course of viral or parasitic infections [35,52] and exposure to cytokines [53]. Consequently, it is possible that the present findings indicate that the underlying mechanisms responsible for presentation of classical MHC I on the cell surface can be linked to a complex cascade of reactions in which other immune molecules and cells may be involved.

Regardless of the initial stimulus, a common response leading to NO synthesis is the upregulation of inflammatory cytokines such as interferon gamma, a potent MHC I inducer. Following peripheral lesioning, production of NO influences the expression of MHC I in the CNS microenviroment, affecting the synaptic plasticity process. The present results are consistent with this hypothesis, since iNOS^-/- ^mice displayed significantly reduced expression of MHC I one and two weeks after lesioning. This is relevant to the regenerative outcome because C57BL6/J mice, which express reduced amounts of classical MHC I compared to A/J animals, show decreased axonal growth in regenerating peripheral nerve and a delayed return of peripheral nerve function [26].

Along with the neuronal response to injury, the glial reaction also plays an important role in synaptic plasticity of the CNS after injury and, to assess this, actin-binding proteins have previously been used as effective microglial markers in immunohistochemistry [36]. Although the microglial reaction in our study did not differ between the two mice strains, MHC I protein was co-localized with the Iba-1 marker, indicating that microglia upregulate such molecules soon after axotomy. As noted previously, both microglial and astroglial cells may be implicated in synaptic elimination after neural damage [9,34,54,55]. In a previous study, the present authors showed that the increased astroglial reaction may be related to a greater stripping of synaptic boutons found in A/J mice [25]. In this case, the normal activity of iNOS could lead to astrogliosis and the expression of MHC I. Conversely, a milder GFAP expression on both the unlesioned and lesioned sides of iNOS^-/- ^mice, as compared to WT mice (*in vivo *as well as *in vitro*), could contribute to the decreased synaptic retraction observed herein. In fact, iNOS knockout mice showed a significantly decreased number of terminals per 100 μm of motoneuron membrane on the contralateral (unoperated) side, reinforcing the idea that iNOS molecules could be involved in the mechanism of synapse refinement and plasticity.

The results obtained in the present study reinforce the importance of the MHC I expression by neurons and reactive glia after peripheral injury, and indicate that microglial cells respond to most glial expression of such molecules. A role for microglia in the synaptic elimination process has been suggested previously as a result of ultrastructural studies. Nonetheless, secondarily activated astrocytes may contribute to maintenance of the synaptic elimination process in the acute phase after injury, since their cytoplasmic processes interpose between retracted pre-synaptic terminals and the motoneuron cell membrane.

NO production in the CNS is related to an increasingly diverse variety of responses that range from intercellular signaling to the necrotic death of cells and the response to invading pathogens. On the other hand, due to the absence of selective NOS inhibitors, it has been difficult to assign specific roles to the different isoforms. NOS-2, or iNOS, is an enzyme capable of producing a continuous flux of NO in the presence of adequate substrate and co-factors. It is expressed in most cells after induction by immunologic and inflammatory stimuli [56]. The present study demonstrates that sciatic nerve transection induces different levels of H2-D^b ^and -K^b ^expression in C57BL6/J and iNOS^-/- ^mice. The iNOS isoform is expressed by astrocytes, macrophages and microglia, following an immunological or inflammatory stimulation [29,31,57].

## Conclusion

In conclusion, the present study shows that knockout mice for the inducible form of NOS display decreased expression of MHC I after lesioning and greater synaptic elimination after peripheral axotomy. Such changes correlate with increased microglial activity without expression of MHC I, coupled with reduced astroglial reaction. These observations are in line with the greater synaptic loss observed in mutant mice that are unable to express MHC I [23]. Further studies aimed at increasing local iNOS activity may investigate upregulation of MHC I molecules by glial cells, which may increase the possibility of a better regenerative outcome after injury.

## Competing interests

The authors declare that they have no competing interests.

## Authors' contributions

AE designed and performed the experiments, analyzed the data, prepared the figures and wrote the manuscript. GFS contributed to the acquisition and analysis of the data. RGZ performed the western blotting analyses. ALRO conceived and designed the study, analyzed the data and wrote the manuscript. All authors read and approved the final manuscript.
